# Surgical correction for adult spinal deformity increases acetabular lateral coverage of femoral heads

**DOI:** 10.1186/s12891-021-04827-z

**Published:** 2021-11-26

**Authors:** Qiang Luo, Yong-Chan Kim, Ki-Tack Kim, Kee-Yong Ha, Young-Soo Chun, Joonghyun Ahn, Sung-Min Kim, Kyeonguk Min

**Affiliations:** 1 Department of Orthopaedic Surgery, Spine Center, Kyung Hee University Hospital at Gangdong, 892, Dongnam-ro, Gandong-gu, Seoul, 05278 South Korea; 2 Department of Orthopaedic Surgery, Graduate School of Medicine, Kyung Hee University, Kyungheedae-ro, Dongdaemun-gu, Seoul, Republic of Korea

**Keywords:** Adult spinal deformity, Tönnis angle, Lateral center edge angle, Angle of sharp, Pelvic tilt

## Abstract

**Background:**

Studies explaining the relationship between hip and spine reported that spinal corrective surgery affected acetabular orientation and changes in pelvic tilt were capable of influencing radiographic measures of acetabular coverage. This study aimed to assess the change in coronal parameters for acetabular coverage as a result of adult spinal deformity (ASD) correction and to analyze the relationship between the postoperative changes in sagittal spinopelvic parameters and coronal acetabular coverage parameters.

**Methods:**

Fifty-two consecutive patients who had undergone multilevel spinal surgical correction were enrolled and evaluated. Coronal acetabular coverage parameters included Tönnis angle (TA), lateral center edge angle (LCEA), and the angle of Sharp (SA). All radiographic parameters were evaluated at the preoperative and the postoperative 1 year. Paired t test was used to determine whether there were significant changes between the time points. Bivariate correlation and linear regression analysis were used to assess the relationship between the postoperative changes of spinal alignment and acetabular orientation.

**Results:**

The surgical correction resulted in significant decrease of TA, increase of LCEA and SA, respectively (*p* < 0.001). The changes in pelvic tilt (PT) demonstrated weak correlation on TA (β = 0.117, *p* < 0.001 for right; β = 0.111, *p* < 0.001 for left).

**Conclusions:**

Although the surgical correction of ASD significantly changed PT resulting in increased acetabular lateral coverage parameters, the correlation between the changes of PT following sagittal correction of ASD and acetabular coverage parameters was low.

**Trial registration:**

This study was retrospectively registered with approval by the institutional review board (IRB) of our institution (approval number: KHNMC-2020-10-010).

## Background

Adult spinal deformity (ASD), regardless of its subtype, is usually characterized by a tendency of bending upper body forward or kyphotic deformity [1]. Fortunately, to some extent, the spinal kyphotic deformity in standing human body can be compensated via hip extension and its resultant change in pelvis which is called pelvic tilt (PT). On the contrary, if the spinal sagittal malalignment is recovered by a corrective surgery, PT as a compensatory mechanism is expected to be changed in a return to its normal position. Thus, the orientation of acetabulum in the pelvis can be also expected to be changed by a corrective surgery on spinal column with sagittal malalignment. In the recent decade, the studies explaining the relationship between hip and spine has been reported that spinal corrective surgery affected acetabular orientation including change of anteversion [2–4]. In addition, Watanabe et al. demonstrated that PT increased in patients with decreased lumbar lordosis (LL) and acetabular coverage of anterior femoral head decreased compared to the controls [5]. Therefore, kyphotic spinal deformity causing excessive PT can substantially contribute to the instability or the risk of dislocation following total hip arthroplasty (THA). In THA for patients with ASD, the planned anteversion and inclination should be less than the native anatomy to prevent risk of anterior instability. Previous studies reported changes in PT could significantly influence radiographic measures of acetabular coverage in cadaveric models [6–8].

Although the correlations were identified in previous cadaveric studies or clinical studies, there had been a paucity of reports clarifying how sagittal deformity correction for patients with ASD significantly increase the acetabular coverage by decrease in PT, increase in LL and consequently alter 3 radiographic measures of acetabular anatomy such as lateral center edge angle (LCEA) [9], Tönnis angle (TA) [10], and the angle of Sharp (SA) [9] and quantifying the relative radiographic changes between the parameters. Therefore, this study aimed to assess and quantify the change in coronal parameters for acetabular coverage as a result of ASD correction and analyze the relationship of postoperative changes of sagittal spinopelvic parameters and the coronal parameters for acetabular coverage. We hypothesized that an alteration in PT following ASD correction would produce significant and predictable differences in the measure of the LCEA, TA and SA. A further understanding of these radiographic relationship may improve surgical plan for patients with concomitant pathologies on both hip and spine.

## Methods

### Study design & patient population

After obtaining approval (approval number: KHNMC-2020-10-010) by the appropriate ethics committee (institutional review board of our institution), a retrospective review of radiographic and clinical data was performed in accordance with the ethical standards laid down in the 1964 Declaration of Helsinki. This study was performed with adult spinal deformity (ASD) patients who had undergone spinal surgical correction between March 2011 and May 2018 at a single institution. The inclusion criteria were as follows: (1) preoperative diagnosis of degenerative lumbar kyphosis or kyphoscoliosis; (2) completion of a long-segment spinal fusion surgery from the level of thoracolumbar junction (T9 to L1) to the sacrum with bilateral S1 pedicle screws and iliac screws for lumbosacral fixation; and (3) postoperative follow-up period of more than 1 year. The exclusion criteria were as follows: (1) sagittal balance as sagittal vertical axis (SVA) less than 5 cm and PT less than 20° on lateral radiograph in standing position; (2) inadequate visibility for measuring radiographic parameters in whole spine standing anteroposterior (AP) and lateral radiographs at regular pre- and postoperative visits; (3) radiographic evidence of osteoarthritis as defined by less than 2 mm of remaining joint space or hip dysplasia; (4) preoperative coronal balance (CB) of > 3 cm or leg length discrepancy (LLD) of > 1 cm; (5) peripheral vascular disease; (6) any syndromic, neuromuscular disease; (7) evidence of previous hip surgery; (8) evidence of previous spine fusion surgery; (9) early (within 1 year) postoperative complications requiring revisional operation for index surgery; (10) if they lacked either baseline or postoperative imaging at regular follow-up; and (11) if there was an obstruction of the normal acetabular ellipse.

### Radiographic assessment

Standing 36-in.-long cassette AP and lateral radiographs of the whole spine were measured preoperatively and at 1-year postoperative follow-up, respectively. On the radiographs, sagittal vertical axis (SVA): the distance between the C7 plumb line and the posterior-superior corner of S1; cervical lordosis (CL) [11]: the angle between the inferior C2 endplate and the C7 endplate; thoracic kyphosis (TK) [11]: the angle between the upper endplate of the T5 vertebra and the lower endplate of the T12 vertebra; thoracolumbar kyphosis (TLK) [11, 12]: the angle between the upper endplate of the T10 vertebra and the lower endplate of the L2 vertebra; lumbar lordosis (LL) [11]: the angle between the superior L1 endplate and the S1 endplate; PT [11]: the angle between the vertical line and the line joining the middle of the sacral plate and the hip axis; and pelvic incidence (PI) [11–13]: the angle between the perpendicular of the sacral plate and the line joining the middle of the sacral plate and the hip axis; were measured. The angle was defined as positive if the curve is kyphotic and as negative if the curve is lordotic. We also performed standard coronal measurements of spinopelvic parameters using standing anteroposterior radiographs: coronal balance (CB) [12]: lateral distance of the C7 plumb-line to the center sacral vertical line; and pelvic obliquity (PO) [12].

**Fig. 1:**
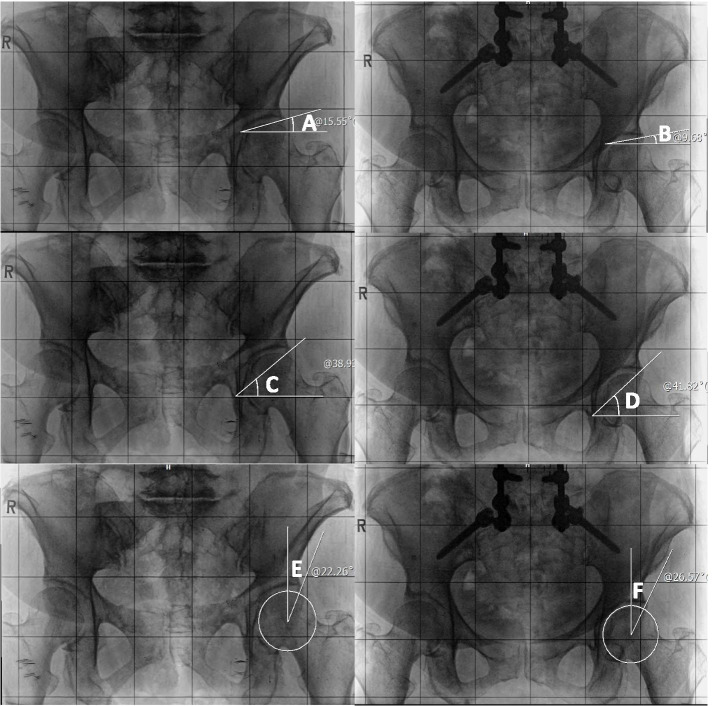
Posterior-anterior pelvic radiographs of a 72-year-old female patient showing the changes in acetabular coverage after surgical correction for ASD. Note that TA decreased after surgery. On the other hand, SA and LCEA increased. **A** Preoperative TA left. **B** Postoperative TA left. **C** Preoperative SA left. **D** Postoperative SA left. **E** Preoperative LCEA left. **F** Postoperative LCEA left

.Coronal acetabular coverage parameters included TA, LCEA, and SA (Fig. 1). The TA was measured between a horizontal reference and a line formed parallel to the most medial and lateral extents of the sclerotic weight bearing portion of the acetabulum [6]. The LCEA was calculated by superimposing a circle over the acetabulum and measuring the angle between a vertical reference and the lateral edge of the sourcil with the apex at the center of the superimposed circle [6]. The SA’s apex was centered at the inferior radiographic teardrop and measured between a vertical reference and the lateral acetabular rim [6]. These coronal acetabular parameters were measured on standing, posterior-anterior, 36-in. long radiograph. Of note, the protocol for image acquisition called for a weight-bearing, freestanding, comfortable position with arms flexed at 45 degrees to avoid superimposition with the spine [14].

### Inter- and intraobserver reliability

Intraclass Correlation Coefficients for acetabular lateral coverage parameters were calculated within and between the observers by selection of randomly selected 20 patients among included patients. All measures were independently acquired and recorded by 2 observers (JA, SMK). The measures were taken at 2 separate time points, a minimum of 2 weeks apart. To reduce random error, then, the measured values were averaged for statistical analysis with spinopelvic parameters.

### Statistical analysis

All statistical analysis were performed with SPSS software (version 21.0, Armonk, NY, USA). Complete-case analysis was used and data were summarized by mean ± standard deviation (SD) or range for numeric variables. Distribution normality was assessed using the Kolmogorov-Smirnova test. Paired t test was used to compare the measured values between pre- and postoperative spinopelvic and acetabular coverage parameters. Jonkheere-Terpstra test was used to compare the coronal acetabular coverage parameters (TA, LCEA, and SA) among three groups (Group I: PI < 45, Group II: PI 45–60°, Group III: PI > 60°) for both sides of hip. The effect of change in spinopelvic parameters on the change of coronal acetabular coverage parameters was determined by bivariate correlation analysis and linear regression analysis. Statistical significance was set at *P* < 0.05.

## Results

### Demographics and clinical details

Among the 144 consecutively treated ASD patients, a total of 52 patients (104 acetabulum) met the study inclusion criteria. There were 42 women (86.1%) and 10 men (13.9%). The age of patients was 69.5 ± 3.8 (range, 62–77) years and body mass index (BMI) was 23.2 ± 5.6 (range, 16.9–35.2) kg/m^2^. The demographic and clinical data were further summarized in Table 1.

**Table 1:** Demographic Characteristics of the Patients

Gender	42 women; 10 men
Age (years)	69.5 ± 3.8 (range, 62–77)
BMI (kg/ m^2^)	23.2 ± 5.6 (range, 16.9–35.2)
Diagnosis	
Degenerative lumbar kyphosis	34 cases
Degenerative lumbar kyphoscoliosis	18 cases
Number of fusion levels	8.0 ± 0.6 (range, 6–9)
T9 to Sacrum(9 levels)	8 cases
T10 to Sacrum(8 levels)	40 cases
T11 to Sacrum(7 levels)	2 cases
T12 to Sacrum(6 levels)	2 cases

*BMI* Body Mass Index.

The data in the table are presented as mean ± standard deviation or range.

### Repeatability and reproducibility of measurement

Intra- and interobserver correlation coefficients for each measure of acetabular lateral coverage parameters ranged between 0.86 and 0.92, and 0.87 and 0.96 for each observer and separate time point, respectively (Table 2).

**Table 2:** Intra- and Interobserver agreement

Variable	Intra-observer ICC (95% CI)	Inter-observer ICC (95% CI)
TA	0.92 (0.79–0.97)	0.87 (0.76–0.95)
SA	0.86 (0.65–0.95)	0.96 (0.90–0.98)
LCEA	0.92 (0.81–0.97)	0.89 (0.71–0.96)

*ICC* Intraclass correlation coefficient, *CI* Confidence interval, *TA* Tönnis angle, *LCEA* Lateral center edge angle, *SA* Angle of Sharp.

ICC calculated using the 2-way-random effects model.

### The changes of spinopelvic parameters after ASD correction

Significant sagittal radiographic correction was achieved with surgery for SVA, CL, TLK, LL, PT, and PI-LL (*P* < 0.05). The spinopelvic alignment was significantly improved at 1 year postoperatively. SVA, PI-LL, and PT decreased significantly from baseline to the 1-year postoperative follow-up (SVA: [13.6 ± 6.4 to 3.7 ± 2.2, *p* < 0.001], PI-LL: [37.1 ± 16.2 to 9.6 ± 5.5, *p* < 0.001], PT: [32.9 ± 8.1 to 20.4 ± 5.7, *p* < 0.001]). LL, SS, and TK increased from baseline to the 1-year postoperative follow-up (LL: [15.4 ± 14.5 to − 44.4 ± 7.7, *p* < 0.001], SS: [22.0 ± 9.0 to 32.4 ± 8.7, *p* < 0.001], TK: [13.1 ± 11.7 to 26.5 ± 8.7, *p* < 0.001]). In addition, the absolute value of coronal radiographic measurements such as CB and PO decreased significantly after surgery (CB: [10.3 ± 8.6 to 3.6 ± 2.8, *p* < 0.001], PO: [1.0 ± 0.9 to 0.6 ± 0.6, *p* = 0.002]). These changes of spinopelvic parameters after ASD correction are summarized in Table 3.

**Table 3:** The changes of spinopelvic parameters after ASD correction

	Value mean ± SD	*P*		Value mean ± SD	*P*
**Sagittal parameters**					
SVA (cm)			LL (°)		
Preop	13.6 ± 6.4		Preop	15.4 ± 14.5	
Po 1Y	3.7 ± 2.2	< 0.001*	Po 1Y	− 44.4 ± 7.7	< 0.001*
SS (°)			PT (°)		
Preop	22.0 ± 9.0		Preop	32.9 ± 8.1	
Po1Y	32.4 ± 8.7	< 0.001*	Po 1Y	20.4 ± 5.7	< 0.001*
TK (°)			PI (°)		
Preop	13.1 ± 11.7		Preop	53.9 ± 9.0	
Po 1Y	26.5 ± 8.7	< 0.001*	Po 1Y	54.2 ± 9.0	0.143
TLK (°)			PI-LL (°)		
Preop	8.6 ± 12.1		Preop	37.1 ± 16.2	
PO 1Y	7.5 ± 8.2	0.458	Po 1Y	9.6 ± 5.5	< 0.001*
**Coronal parameters**					
CB(mm)			PO (°)		
Preop	10.3 ± 8.6		Preop	1.0 ± 0.9	
Po 1Y	3.6 ± 2.8	< 0.001*	Po 1Y	0.6 ± 0.6	0.002*

*Preop* Preoperative, *Po 1Y* 1-year postoperative, *SVA* Sagittal vertical axis, *SS* Sacral slope, *TK* Thoracic kyphosis, *TLK* Thoracolumbar kyphosis, *LL* Lumbar lordosis, *PT* Pelvic tilt, *PI* Pelvic incidence, *CB* Coronal balance, *PO* Pelvic obliquity, *SD* Standard deviation.

The data in the table are presented as the mean ± SD.

P, Comparison between preoperative and postoperative outcomes using paired t test.

* Statistically significant *(p < 0.05)*

### The changes of radiographic parameters for acetabular coverage after ASD correction

The surgical correction for ASD resulted in a significant decrease of TA (*p* < 0.001), increase in LCEA (*p* < 0.001) and SA (*p* < 0.001), on both sides, respectively (Table 4) (Fig. 1).

**Table 4:** Comparison of radiographic acetabular parameters between pre- and postoperative values

	Right Side		Left Side	
	Value mean ± SD	*P*	Value mean ± SD	*P*
TA (°)				
Preop	7.5 ± 2.4		7.3 ± 2.7	
Po 1Y	5.7 ± 1.7	< 0.001*	5.5 ± 1.8	< 0.001*
LCEA (°)				
Preop	33.1 ± 4.7		35.1 ± 5.3	
Po 1Y	37.3 ± 4.8	< 0.001*	38.5 ± 4.9	< 0.001*
SA (°)				
Preop	37.1 ± 3.3		36.5 ± 3.5	
Po 1Y	39.1 ± 3.4	< 0.001*	38.8 ± 3.8	< 0.001*

*Preop* Preoperative, *Po 1Y* 1-year postoperative, *TA* Tönnis angle, *LCEA* Lateral center edge angle, *SA* Angle of Sharp.

The data in the table are presented as the mean ± SD.

P, Comparison between preoperative and postoperative outcomes using paired t test.

*, statistically significant *(p < 0.05)*

### Comparison of the change of acetabular coverage after ASD correction according to PI

We further evaluated the difference in outcomes by dividing the patients into 3 subgroups according to PI: Group I, PI ≤45°, Group II, 45° < PI ≤60°, and Group III, PI > 60° (Table 5). Although there was statistically significant decrease of TA, increase of LCEA and SA within each group after ASD correction, there were no significant differences among the subgroups by PI except preoperative TA right side, (*p* = 0.044), the 1-year postoperative LCEA right side (*p* = 0.045) and LCEA right side (*p* = 0.002).

**Table 5:** Comparison of the change of acetabular coverage after ASD correction according to PI

	Group I (PI < 45°)	***P***	Group II (PI 45–60°)	***P***	Group III (PI > 60°)	***P***	Jonckheere-Terpstra test	Mann-Whitney U test
TA (°)								
Right Side								
Preop	9.0 ± 3.7		7.0 ± 1.8		7.9 ± 3.0		0.339	0.321
Po 1Y	7.4 ± 2.4	< 0.001*	5.5 ± 1.4	< 0.001*	5.4 ± 1.6	< 0.001*	0.044*	0.059
Left Side								
Preop	9.2 ± 4.3		6.6 ± 1.7		8.1 ± 3.4		0.660	0.541
Po 1Y	7.3 ± 2.7	< 0.001*	5.2 ± 1.4	< 0.001*	5.4 ± 1.6	< 0.001*	0.108	0.093
LCEA (°)								
Right Side								
Preop	31.2 ± 4.3		34.1 ± 4.6		31.0 ± 4.8		0.578	0.888
Po 1Y	33.1 ± 1.6	< 0.001*	38.3 ± 5.2	< 0.001*	37.4 ± 2.9	< 0.001*	0.045*	0.002*
Left Side								
Preop	32.0 ± 4.7		36.2 ± 5.4		33.2 ± 3.8		0.863	0.541
Po 1Y	35.0 ± 2.7	< 0.001*	39.4 ± 5.3	< 0.001*	38.1 ± 3.7	< 0.001*	0.174	0.139
SA (°)								
Right Side								
Preop	37.0 ± 1.3		37.1 ± 3.8		37.2 ± 2.5		0.878	0.888
Po 1Y	38.8 ± 1.6	< 0.001*	38.9 ± 3.8	< 0.001*	40.1 ± 2.8	< 0.001*	0.479	0.277
Left Side								
Preop	36.5 ± 2.6		36.5 ± 3.9		36.4 ± 3.3		0.939	1.000
Po 1Y	40.0 ± 2.8	< 0.001*	38.6 ± 4.0	< 0.001*	39.4 ± 4.1	< 0.001*	0.619	0.743

*PI* Pelvic incidence, *Preop* Preoperative, *Po 1Y* 1-year postoperative, *TA* Tönnis angle, *LCEA* Lateral center edge angle, *SA* Angle of Sharp.

The data in the table are presented as the mean ± SD.

P, Paired-t test between Preop and Po 1Y.

Jonckheere-Terpstra test, among Group I, II and III.

Mann-Whitney U test, between Group I and Group III.

* Statistically significant if *p < 0.05*

### Correlations between spinopelvic parameters and acetabular parameters

**Fig. 2:**
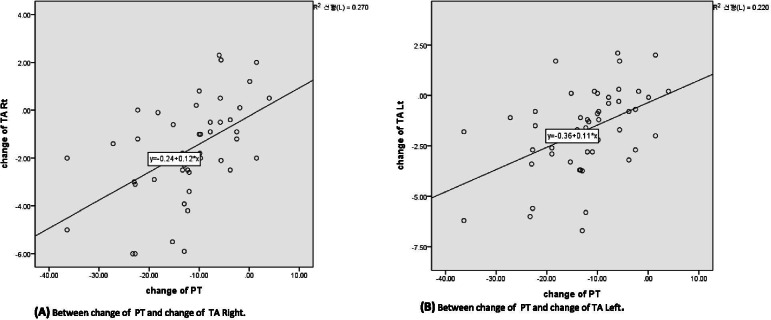
Graphs of Scatterplot explaining the linear regression between the changes of PT and TA (right and left) after ASD correction. **A** The relationship between the change of PT and the change of TA right. **B** The relationship between the change of PT and the change of TA left

As shown in Table 6, the results from Pearson bivariate correlation analysis between spinopelvic parameters and coronal acetabular coverage parameters revealed that PT (vs TA right: r = 0.520, *p* < 0.001; vs TA left: r = 0.469, *P* < 0.001), LL (vs LCEA right: r = 0.335, *P* = 0.015), and TK (vs TA left: r = 0.289, *P* = 0.038) significantly correlated with coronal acetabular coverage parameters. With stepwise linear regression analysis, it was revealed that 1° decrease of PT was occurred with 0.117° decrease of TA right (R^2^ = 0.270), and with 0.111° decrease of TA left (R^2^ = 0.220) (Table 7) (Fig. 2).

**Table 6:** Bivariate correlation analysis between the radiographic change of spinopelvic parameters and acetabular coverage parameters after ASD correction

Change of		TA		LCEA		SA	
		Right	Left	Right	Left	Right	Left
**TK**	*r* *p*	−.254 (.069)	−.289^*^ (.038)	.155 (.274)	.154 (.275)	−.154 (.275)	−.019 (.894)
**TLK**	*r* *p*	.029 (.836)	.034 (.809)	.043 (.760)	.070 (.621)	.207 (.140)	.134 (.345)
**LL**	*r* *p*	−.223 (.112)	−.171 (.225)	.335^*^ (.015)	.150 (.289)	−.021 (.882)	−.123 (.384))
**PT**	*r* *p*	.520^***^ (<.001)	.469^***^ (<.001)	−.202 (.151)	−.271 (.052)	−.194 (.168)	.035 (.804)
**SS**	*r* *p*	−.189 (.178)	−.165 (.242)	.170 (.228)	.155 (.272)	.049 (.732)	.037 (.793)
**PI-LL**	*r* *p*	.262 (.061)	.210 (.135)	−.309^*^ (.026)	−.080 (.571)	.066 (.641)	.208 (.138)

*TA* Tönnis angle, *LCEA* Lateral center edge angle, *SA* Angle of Sharp, *TK* Thoracic kyphosis, *TLK* Thoracolumbar kyphosis, *LL* Lumbar lordosis, *PT* Pelvic tilt, *SS* Sacral slope, *PI* Pelvic incidence, *r* Pearson correlation coefficient

*, p < .05; **, p < .01; ***, p < .001

*p < 0.05*, statistically significant (correlation).

**Table 7:** Linear regression analysis of radiographic parameters predicting the changes of TA and PT after ASD correction

	*B*	SE	*P*-value	R^2^
Change of TA Right				
Constant	−.243	.413		
Change of PT	.117^***^	.027	<.001	.270
Change of TA Left				
Constant	−.360	.445		
Change of PT	.111^***^	.029	<.001	.220

*B* Unstandardized Regression Coefficients, *SE* Standard Error, *R*^*2*^Coefficient of determination.

***, P < .001

*TA* Tönnis angle, *PT* Pelvic tilt.

## Discussion

Our study apparently demonstrated that surgical correction for ASD consequently increased the parameters representing acetabular lateral coverage (decrease of TA, increase of LCEA and SA) on femoral head. However, unlike a similar study on the relationship between acetabular anteversion and spinal deformity correction [3], we could not find a correlation coefficient with statistical significance between the change of PT and other acetabular coverage parameters such as LCEA and SA. In our study, the result from bivariate analysis between spinopelvic parameters and acetabular coverage parameters revealed that change of PT significantly correlated with change of TA. In explanation with linear regression model, although the correlation between TA and PT was low, postoperative TA decreased simultaneously with postoperative decrease of PT compared to their preoperative values.

One way to maintain this spinopelvic alignment is to retrovert the pelvis (increase of PT) that may be seen as a backward rotation of the pelvis around the hips [15]. From the perspective of compensatory mechanism for upright posture and horizontal gaze in human, we can infer the acetabular orientation on the basis of the relationship between the spine and the pelvis. When pelvis rotates anteriorly in an increased posterior tilted pelvis, acetabular coverage increased along with decreased PT because the acetabulum is a deep, cup-shaped structure that is normally oriented to face forward and outward, three-dimensionally [8]. Based on these findings, we hypothesized that correction of ASD with increased PT might result in the decrease of PT, and the increase of anterior and lateral acetabular coverage as well. Although TA, LCEA, and SA are anatomical constant parameters of acetabulum, the result of this study demonstrated that surgical correction of ASD was capable of creating statistically significant changes in radiographic measures of TA, LCEA, and SA which were coronal parameters to present acetabular coverage [6]. On the whole, the results of this study reinforced previous studies which reported on the impact of the change of PT on acetabular coronal radiographic measures [13, 16].

In another study on the relationship between spinopelvic alignment and acetabular coverage, the measures of LCEA were found to have weak inverse correlation with LL but poor correlation with PI and PT. [17] Moreover, they added TA was not correlated with any of sagittal spinopelvic measurements [17]. According to the recent research by McQuivey et al., higher TA (> 10 degrees) portended a higher risk for revision surgery after arthroscopic surgery on mild hip dysplasia [18]. We also think this change of TA acquired by ASD correction might have an effect on further development of hip pathology. However, on the evidence of the partial discordance between the prior literatures and our study, we couldn’t absolutely conclude the universal relationship between the changes of the investigated acetabular parameters and PT. Nonetheless, it is apparent that corrective surgery of patients with ASD can increase LL and decrease PT, and lead to a consistent change of acetabular orientation and lateral coverage.

Above the things aforementioned, the most important clinical implication of the changed acetabular coverage and orientation is thought to focus on the pathogenesis of hip disease, and the correlation with THA [19]. A few studies investigated on the relationship between sagittal pelvic malrotation by PT and THA [20–24]. Tang et al. demonstrated that sagittal pelvic malrotation may potentially lead to the malpositioning of acetabular components despite the careful intraoperative verification of the correct placement [22]. Lazennec et al. reported that patients with spinal fusion demonstrated less adaptability of the lumbosacral junction and longer spinal fusion or inclusion of the pelvis in the fusion critically impacted hip-spine biomechanics and significantly affected the ability to compensate in the standing-to-sitting transition [23]. Buckland et al. reported that since the patients with spinopelvic malalignment had a high prevalence of excessively anteverted acetabular position, sagittal spinal correction following THA resulted in reduced acetabular anteversion, which may have implication for permissible instability [3]. Furuhashi et al. reported spinal long fusion with pelvic fixation could be a risk factor for posterior THA dislocation [24]. However, some authors reported sagittal imbalance did not influence cup anteversion in THA, and therefore the dislocation might not be correlated with spinal sagittal imbalance [25]. If a patient with previous history of THA undergo spinal deformity correction, it is expected that the increased PT as a compensatory mechanism will decrease postoperatively and normalize the anterior and lateral acetabular coverage on femoral head, and which can improve the anterior stability of hip joint. However, in sitting position (flexion of hip joint), it is apprehended that the excessive increase of LL and SS by spinal correction might cause decreased PT that jeopardize posterior dislocation of THA. Meanwhile, when a patient with severe kyphosis and excessively increased PT is planned to undergo THA, the coverage of acetabular component on femoral component is still expected to be deficient postoperatively. Theoretically, it is thought that consistent eccentric joint reaction force affects in THA site. Therefore, we believe it is also worth investigating the potential instability, dislocation, polyethylene wear in patients of ASD with increased PT.

The ability of PT is limited by the value of person’s own PI. Patients with a small PI have a small capacity to compensate for their sagittal imbalance through pelvis retroversion [26]. Therefore, we initially postulated that the patients with higher PI would have more postoperative decrease in PT and resultant increase in acetabular coverage parameters. However, subgroup analysis according to PI showed that significant differences were found only in the preoperative TA on the both sides and the ultimate postoperative LCEA on the right side. Although there was no significant difference, the patients with high PI (> 60°) demonstrated a trend toward the higher TA. This result was thought to imply that the higher PI show the more preoperative tendency to have acetabular orientation to decrease anterior and lateral coverage because they recruited more PT preoperatively as a compensatory mechanism. We think further study with large population is warranted to clarify the relationship between PI and postoperative change of acetabular coverage parameters following ASD correction.

In this study, to focus on the spinal sagittal correction of kyphosis and reduce confounding factors (coronal imbalance or functional scoliosis due to LLD), we excluded the patients who preoperatively showed an LLD of > 10 mm or a CB of > 3 cm in whole spine standing AP radiographs. The decision of the exclusion criteria on coronal balance was based on previous literatures [27–30]. Khamis and Carmeli recently reported that an LLD of > 10 mm could generate substantial changes in gait, with greater differences in leg length having greater impact [27]. Radcliff et al. found an association between pelvic obliquity as a result of LLD and degenerative scoliotic curve morphology in patients undergoing lumbar fusion for the treatment of degenerative scoliosis or degenerative spondylolisthesis [30]. Furthermore, since acetabular orientation was delicately affected by whether weight bearing was applied or not, all the radiographic measures in this study were taken under weight bearing position [30].

There are several limitations in this study. Although correction of ASD improved acetabular coverage parameters with significant changes, we could not directly measure acetabular anteversion, which was considered as a more important parameter for the clinical aspect of hip joint motion. And the clinical outcomes after ASD correction to reveal the clinical relevance to the changes in acetabular coverage parameters were not included. If the comparison between the patients with and without THA was conducted, it would have provided additional clinical implications. Despite the effort to reduce confounding influences on each measurement, PO or LLD were considered to have debilitate the relationship between the change of acetabular coverage parameters and the change of PT achieved by ASD correction due to measurement of acetabular coverage parameters taken not by standing pelvis radiographs but by standing whole spine AP long radiographs.

## Conclusions

This study found that the correlation between the change of PT and coronal acetabular coverage parameter was low although sagittal correction of ASD significantly changed acetabular orientation resulting in increased lateral coverage parameters. However, it is expected that preoperatively increased PT will decrease postoperatively and its resultant increase of anterior and lateral acetabular coverage on femoral head may provide better anterior and lateral stability on hip joint.

## Data Availability

The patients’ data were collected in Kyung Hee University Hospital at Gangdong. The datasets generated and/or analysed during the current study are available from the corresponding author (JA) on reasonable request.

## Abbreviations

ASD: Adult spinal deformity
TA: Tönnis angle
LCEA: Lateral center edge angle
SA: The angle of Sharp
TK: Thoracic kyphosis
TLK: Thoracolumbar kyphosis
LL: Lumbar lordosis
PT: Pelvic tilt
SS: Sacral slope
PI: Pelvic incidence
SVA: Sagittal vertical axis
CB: Coronal balance
LLD: Leg length discrepancy
PO: Pelvic obliquity
AP: Anteroposterior
BMI: Body mass index
THA: Total hip arthroplasty
